# Deep-Learning Based Estimation of Dielectrophoretic Force

**DOI:** 10.3390/mi13010041

**Published:** 2021-12-28

**Authors:** Sunday Ajala, Harikrishnan Muraleedharan Jalajamony, Renny Edwin Fernandez

**Affiliations:** Department of Engineering, Norfolk State University, Norfolk, VA 23504, USA; s.a.ajala@spartans.nsu.edu (S.A.); h.jalajamony@spartans.nsu.edu (H.M.J.)

**Keywords:** dielectrophoretic (DEP), AlexNet, MobileNetV2, VGG19, neural network, textile electrode, pearl chain, convolutional neural networks (CNN), force

## Abstract

The ability to accurately quantify dielectrophoretic (DEP) force is critical in the development of high-efficiency microfluidic systems. This is the first reported work that combines a textile electrode-based DEP sensing system with deep learning in order to estimate the DEP forces invoked on microparticles. We demonstrate how our deep learning model can process micrographs of pearl chains of polystyrene (PS) microbeads to estimate the DEP forces experienced. Numerous images obtained from our experiments at varying input voltages were preprocessed and used to train three deep convolutional neural networks, namely AlexNet, MobileNetV2, and VGG19. The performances of all the models was tested for their validation accuracies. Models were also tested with adversarial images to evaluate performance in terms of classification accuracy and resilience as a result of noise, image blur, and contrast changes. The results indicated that our method is robust under unfavorable real-world settings, demonstrating that it can be used for the direct estimation of dielectrophoretic force in point-of-care settings.

## 1. Introduction

In dielectrophoresis, a nonuniform electrokinetic field is used to apply a force on uncharged or neutral particles resulting in the motion of the particle suspended in a medium by the interactions of this nonuniform electric field and the induced effective dipole moment of the particle [1,2,3]. Dielectrophoretic (DEP) forces assemble particles from aqueous suspensions into long electrically conductive nano/microstructures due to positioning, convection, and levitation. Electric field-induced dipole formation causes pearl chaining of particles that are aligned along an electric field [4,5]. Pearl chains are direct indicators of DEP forces and can be correlated to dielectric variations of a variety of microparticles, including biological cells [1,2,3,6,7].

Despite the fact that dielectrophoresis is becoming increasingly relevant in point-of-care systems [6], DEP force estimation is still theoretical. The computation of the DEP force acting on a particle has been described as a challenging task unless numerous simplifying assumptions and relatively simple geometries are taken into account [4], and these are commonly based on Pohl’s dipole approximation [1].

The three key elements to determine the DEP force are the dielectric properties of the medium, the intrinsic dielectric properties of the particle, and the presence of a nonuniform electric field. The interactions between these elements are used to manipulate and separate particles in a particular medium using DEP forces. In [8,9], two distinct approaches for calculating electric force are provided. The first is based on the equivalent dipole method (EDM), which is simple to apply but inaccurate in some instances [1,9]. For example, the particles could be adjacent to an electrode, or two or more particles could be crowded together. In the EDM approximation, the DEP force is estimated using the electric field without considering the effects of other particles. The second is based on the Maxwell stress tensor (MST) and provides accurate results in all feasible conditions, but it is far more difficult to execute [10]. The MST approach has been used to investigate the interactions of finite-size particles in a DC electric field [10,11,12,13,14,15,16,17] and AC electric field [18,19,20]. However, it is not feasible to study the interactions of multiple particles using the MST method. In this situation, the electric potential around the particle is recreated using the multipolar approximation. Nakajima and Hernandez [21,22] investigated the electric field distribution around the particles of the particle chains using the multipolar approximation method.

An iterative dipole moment method (IDM) was published in [9] to determine the interaction forces and movements of multiple DEP particles in an AC field. In the DC field, the accuracy, convergence, and simplicity of the IDM approach have been demonstrated [23,24,25]. Velocity tracking [26] has been used to quantify the DEP force by monitoring the displacement of oocysts [26] and mitochondria [27] over time under electrodeless DEP in a microfluidic device with insulator constrictions in order to obtain particle velocity and acceleration and, in turn, estimate the DEP force exerted on them.

In recent years, CMOS chips combined with dielectrophoretic cell manipulation have piqued interest, allowing for the simultaneous observation of hundreds of individual cells at point-of-care [28,29,30,31,32]. Various microfluidic devices have evolved driven by the desire to improve mobility and accessibility for point-of-care testing of infectious diseases. A low voltage DEP operation is required to integrate DEP-based cell separation in a handheld point-of-care device. DEP-based cell manipulation and sorting are beneficial for on-chip immunological sensing because they improve the local concentration of target particles via DEP force, improving sensitivity and shortening time. Sapsford et al. [33] used this strategy to create a lab-on-a-chip device for detecting and genetically identifying E. coli and SEB toxin. In [34], the DEP force was used with scanning electron microscopy (SEM) to analyze viable cells in a vaporous atmosphere to capture and immobilize viable cells in the SEM region without using a chemical surface modification.

In the development of high-efficiency microfluidic devices for particle manipulation, the ability to precisely quantify DEP force exerted on particles is crucial. We have adopted a Convolutional Neural Network (CNN) based deep learning approach to estimate the applied DEP voltage from the pearl chain alignment of spherical microparticles. CNN-based deep learning approach has been used in image processing and visual identification [35,36,37,38,39], image super-resolution [40], image segmentation [41,42,43,44], and damage detection [45]. The authors in [44] created a CNN-based image processing method for the detection of bubble patterns in dense bubbly flows using the shadowgraph technique. A noncontact vibration measurement system based on deep learning and optical flow was presented in [45]. This technique uses deep learning for feature extraction and achieves the classification of a selection of effective active pixels used in measuring the vibration frequency in a noncontact vibration sensor.

In developing high-efficiency microfluidic devices for particle manipulation, the ability to precisely quantify DEP force could be crucial. In this paper, we show how deep learning can be used to precisely calculate the DEP force experienced by polystyrene beads in a polar medium. In this study, we have used three pretrained deep neural networks, AlexNet, MobileNetV2, and VGG-19. Using transfer learning, we trained the models to predict the applied voltages from the micrographs of pearl chains of polystyrene beads formed during dielectrophoresis.

## 2. Materials and Methods

**Figure 1:**
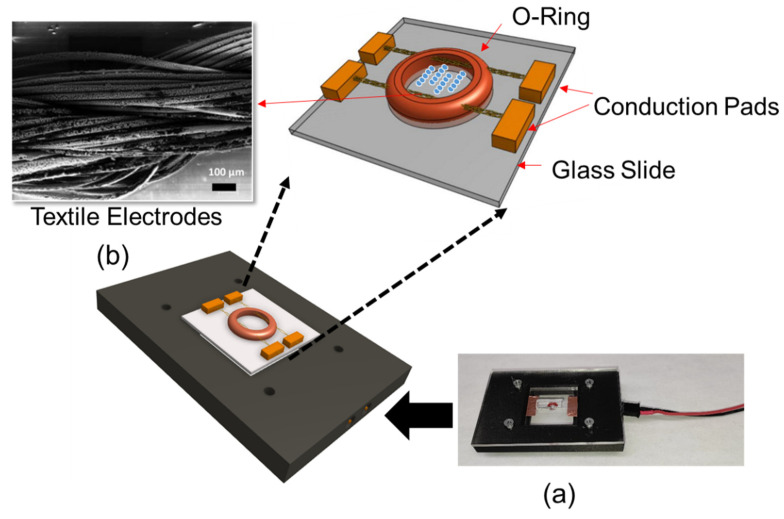
(**a**) Textile electrode-based DEP system with the connection base and (**b**) SEM micrograph of textile electrodes.

The DEP system consisted of textile electrodes sewn through a silicon o-ring (ID: 1 mm, OD: 3 mm). Textile electrodes were silver-coated conductive thread, which was 82% nylon and 18% silver. This structure was mounted on a 1 × 1 inch glass slide. Threads were secured using copper tape, which also acted as an electrical contact. Experiments were performed by introducing 10 µL of liquid sample into the o-ring chamber. The device was enclosed in a 3D printed custom microscope stage for recording images (Figure 1a). Figure 1b shows the Scanning Electron Microscopy (SEM) micrograph of the textile electrodes.

Deep learning analysis was performed using the image datasets obtained from the micrographs of pearl chain formation at various voltages ranging from 1–10 V. For the three experiments, the datasets (700 samples) were partitioned into 80% (i.e., 560 samples) for the training dataset and 20% (i.e., 140 samples) for validation during the training phase of the models. The training was implemented in MATLAB R2021a; we utilized its Deep Learning toolbox for the deep learning experiments. Our development system was a DELL laptop with a five-core Intel 8th Generation processor.

## 3. Theory

### 3.1. DEP Force Estimation

In order to advance DEP aided sensing techniques, a more tangible and straightforward model is required to improve the approximation of the force induced on the particles in terms of the voltage applied. Dielectrophoretic force is the interaction of a nonuniform electric field with a dipole. When the particle is substantially smaller than the nonuniformities in the electric field, the DEP force ($F_{DEP}$) is given by

(1) $$F_{DEP}=2\pi a^{3}\varepsilon_{m}Re\left(\frac{\varepsilon_{p}^{*}-\varepsilon_{m}^{*}}{\varepsilon_{p}^{*}+2\varepsilon_{m}^{*}}\right)\nabla E_{rms}^{2}$$

where a is the radius of spherical particles in a medium of permittivity; $\varepsilon_{m}$ under an ac field; and $\;E_{rms}$, depends on the product of the localized field with its gradient ($\nabla E_{rms}^{2}$) and the frequency-dependent complex dielectric contrast of the particle versus the medium, as given by real-part of the Clausius–Mossoti factor. The relative permittivity of the polystyrene (PS) particle and water medium is set at 2.5 and 78, respectively. $\varepsilon_{p}^{*}$ and $\varepsilon_{m}^{*}$ are the complex permittivities of the particle and the medium, respectively. 

The DEP force resulting from the AC field-induced dipoles in the particles along the electric field causes particle chaining called pearl chains. The DEP force exerted on a particle can be theoretically analyzed using Maxwell–Helmholtz stress method. The electric field and force on spherical dielectric particles can be calculated using this approach. The DEP force on a particle chain where $\varepsilon_{E}$ is the relative permittivity of the surrounding medium, E is the electric field on the particle surface, $E_{n}$ is the normal component of E, and n is the unit normal vector on the surface is given in [46] as:

(2) $$F_{DEP}=\varepsilon_{E}\varepsilon_{0}EE_{n}-\frac{1}{2}\varepsilon_{E}\varepsilon_{0}E^{2}n$$

Various studies [46,47] have indicated that that the force on a chain of spherical dielectric particles in a dielectric fluid is proportional to the number of particles and also the orientation of the chain to the electric field. When the dielectric particles are suspended in a fluid with two 3D parallel electrodes energizing them, the particle chains will be aligned in parallel in the direction of the applied field.

In this study, we focused on predicting the applied voltages by analyzing the orientation of the pearl chains. The deep learning approach predicted the applied voltage on particles from the micrographs obtained. Hence, a direct correlation between the applied voltage and pearl chain formation was established. Our earlier studies have shown that textile electrodes were found to induce strong DEP forces at voltages as low as 4V [48]. However, due to the ambiguities in the thread structure, a theoretical estimation of DEP force from micrographs is difficult. The orientation of textile yarns is significantly different at the micro level. This causes an ambiguity in the estimated force. However, our observations have revealed (a) the average number of particles in a pearl chain, and (b) the alignment of pearl chains to the electric field reflect the dielectrophoretic force on the particle. However, in applications involving large-dimension and large-volume data, extracting manual features is challenging, if not impossible, limiting the capabilities of shallow architectures [44,49]. Deep learning architectures are garnering increasing interest from researchers due to their capacity to learn data representation with several processing layers while also performing automatic feature extraction. As a result, they address the issues that were previously associated with shallow architectures. Deep learning is a framework that aids in solving complex perception tasks with maximum accuracy. It uses nonlinear transformations and model abstractions at a high level for the purpose of conversion and feature extraction [50,51].

### 3.2. Convolutional Neural Network for Deep Learning

Convolutional Neural Network (CNN), also known as ConvNet (Figure S1), is an architecture that employs convolution mathematics to process data with a grid-like topology, such as image data in one, two, and three dimensions. It was primarily created to adapt the neural network to the challenges of image processing, but it may also be applied for other types of data with spatial, temporal, or both dimensions [49,51]. CNNs are specialized for processing data in a known grid-like spatial structure with a stack of convolutions and nonlinear activation functions. The convolution of an image matrix i having dimensions (P, Q) with the kernel h having dimensions (U, V) results in an output matrix o is given by:

(3) $$o\left[i,j\right]=\overset{U-1}{\underset{u=0}{\sum }}\overset{V-1}{\underset{v=0}{\sum }}h\left[u,v\right]*i\left[p-u,q-v\right]\;$$

where 0≤i<U+Q−1 and 0≤j<V+P−1.

Rectified linear units (ReLU) is the nonlinearity activation function used to learn more complex target functions that vary in a non-linear fashion with inputs, which is given by

(4) R(y)=max(0,y)

where y=Wx+b. ReLU maps positive inputs to a and negative inputs to 0. ReLU is faster to train and reduces the likelihood of a vanishing gradient, unlike the sigmoid and hyperbolic tangent function. A pooling, or downsampling layer, reduces the spatial size of the activation maps, minimizes the likelihood of overfitting, and provides invariance to local translation. [41].

SoftMax, a Max pooling method, is used at the final output layer to convert the outputs to a probability distribution. The SoftMax method is defined as:

(5) $$S{\left(z\right)}_{j}=\frac{\exp\left(z_{j}\right)}{\sum_{k=1}^{K}\exp\left(z_{k}\right)}\;\;\;for\;\;j=1,\ldots ,K\;$$

AlexNet [50], VggNet [52], and MobileNet [53,54] are the pretrained CNN models examined in this research. With a 1000 weight SoftMax, AlexNet has five convolutional layers, three max pooling layers, dropout, and three fully connected layers. AlexNet’s nonlinearity is achieved using overlap pooling and ReLU. The VggNet uses max pooling to reduce network volume and finishes with two fully connected layers and a SoftMax classifier. It is widely used as a starting point feature extractor for more complex networks. However, the vast size of VggNet (113 million parameters) is a disadvantage.

MobileNet is a CNN network that is well-suited to embedded and mobile vision applications [45]. In comparison to VggNet, it is more accurate and consumes less computing energy. A 3 × 3 depthwise separable convolution, batchnorm, and ReLU nonlinearity are the foundations of the MobileNet architecture. It has shown good promise in the areas of object recognition and detection, geolocalization, facial characteristics, and embedded systems.

Model parameters (weights and biases) are defined using interactive visualizations to minimize the cost function J over the full training dataset, as illustrated in Equation (6):

(6) $$J=\frac{1}{m_{b}}\sum_{i=1}^{m_{b}}L^{\left(i\right)}$$

where $m_{b}$ is the size of the training data, referred to as the mini-batch size for each iteration, and $L^{\left(i\right)}$ is the loss obtained for a single training example $x^{\left(i\right)}$ labeled. Notably, hyperparameter values such as the learning rate α and batch size $m_{b}$ were appropriately selected as shown in Table 1 to achieve a lower cost function and fast optimization while allowing model convergence to the global minimum, thereby reducing overshoots [55]. The mini-batch size was set to a small value for better training accuracy and faster updates, as larger values cause the models to overfit and not generalize.

The optimizer, root mean square propagation (RMSProp), was used to compute the activation of the neurons to test the efficiency of the CNN models [55]. The update rule, as shown in Table 2, shows how the gradients are calculated for each model.

## 4. Results and Discussion

### 4.1. Pearl Chain Formation Using Thread-Based Electrodes

**Figure 2:**
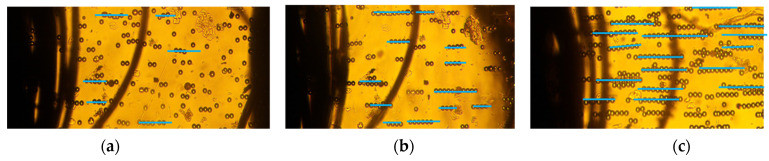
Pearl chain formation of PS microbeads due to positive DEP in low conductivity solution (0.001 mS/m) in thread-based electrodes at (**a**) 1 V, (**b**) 3 V, and (**c**) 6 V @2 kHz.

Our approach combined deep learning algorithms with the results from a series of experiments on 10–20 µm PS microbeads in order to investigate the alignment and movement of particles in static conditions. We investigated the particle chain formation due to the applied electric field at 2 kHz in a low conductivity solution (0.001 mS/m) which invoked a strong positive-DEP (p-DEP). Figure 2a–c depicts the pearl chain formation of microbeads in the textile electrode system at 2 kHz at varying applied voltages. The effect of voltages on the particle chain alignment were examined for different voltages between 1–6 Vpp. The textile electrode pair was at a separation distance of 1 mm. The polystyrene microbeads suspended were aligned in pearl chains. The strength of the electric field was visually apparent from the alignment of the pearl chains. Although particle aggregation depends on the initial position of the particles, the particles always coordinate in a line when the force equilibrium is attained. Hence, any particle can be treated as a representative model in a long pearl chain. Single microspheres were found to be more likely to join an existing pearl chain rather than starting a new pearl chain. This has been attributed to the increase in the magnitude of the electric field gradient with the shortening gap between the pearl chain leading edge and the adjacent electrode. It was also observed that the majority of the particles moved only on the initial plane in a perpendicular electric field. Moreover, the particle alignment varied when particles were further away from the electrodes.

The number of particles varied from 2 to 18 when the voltages varied from 1 to 6 Vpp. Figure 2a depicts the particle orientation at an applied voltage of 1 Vpp. The DEP force was extremely low, and the particles were disoriented and did not form pearl chains. However, at 3Vpp, the particles started to align, and the pearl chains formed had 2–3 particles (Figure 2b). Apparently, the pearl chain alignment was highly parallel at voltages of 6Vpp (Figure 2b) and did not significantly increase with applied voltages. This can be attributed to the large surface area of the textile electrodes, which were around 10^5^ times higher than planar electrodes [48]. At higher volume fractions, lateral interactions between the PS particles and the pearl chains assembled into two-dimensional (2D) arrays.

### 4.2. Deep Learning Model for Image Classification

**Figure 3:**
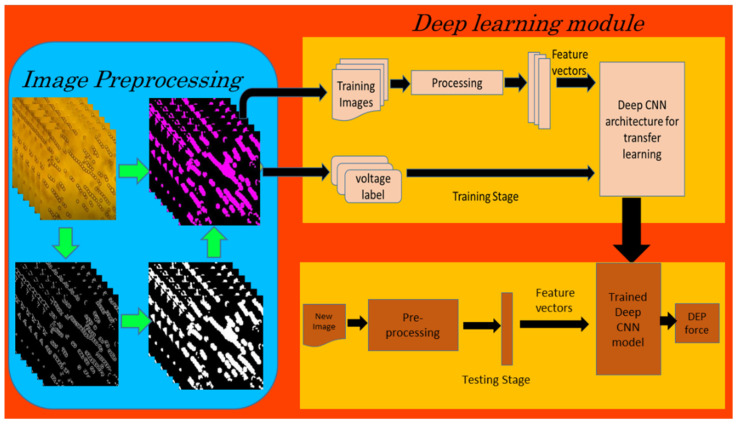
Block diagram of image classification using deep learning (CNN).

After resizing the images to fit the input layer dimension for each model, the image datasets were used to train and validate the deep learning models (Figure 3). AlexNet, VGGNet19, and MobileNetV2 were the three deep CNN models used in our study. A MATLAB script was written to explore the Sobel edge detection algorithm for the image preprocessing tasks, such as the conversion of images from RGB to grayscale, thresholding and edge detection resulting in binary gradient mask, dilation of the binary mask using the vertical and horizontal structuring elements, filling of holes and clearing the border around the peal chains, and reconstruction of the one-channel binary image into a 3-channel RGB image.

### 4.3. Model Testing

**Figure 4:**
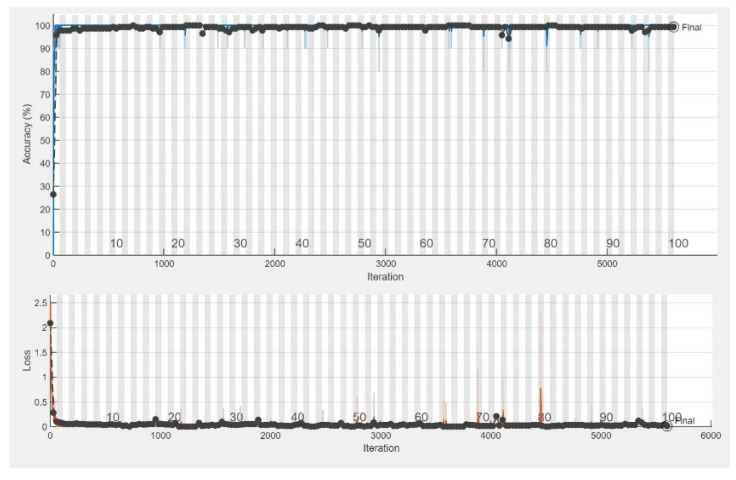
Training progress showing the validation accuracy of MobileNetV2.

**Figure 5:**
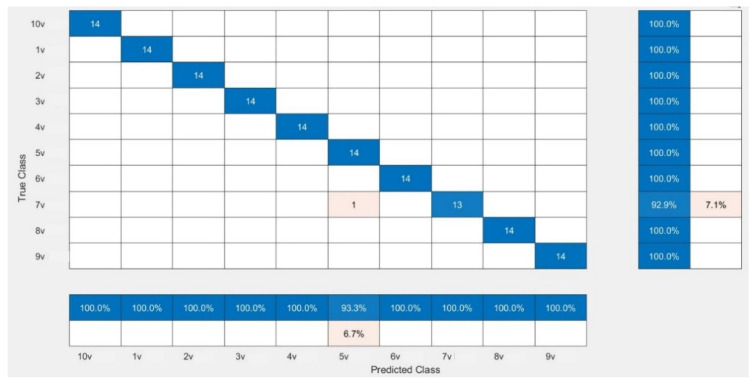
Confusion matrix showing the validation performance of MobileNetV2.

The CNN models were assessed for their performance and validation accuracy to correlate micrographs to input voltages to find the model with the best performance. Each model was trained in 100 epochs with 56 iterations per epoch. Table 3 shows the performance comparison between the pretrained CNN models examined in this study for the first experiment using the dataset. The results from the experiment showed that MobileNetV2 with RMSPROP had the best performance, with a validation accuracy of 99.29%, followed by AlexNet with a validation accuracy of 98.57%, while VGG19 had a lower validation accuracy of 97.86%. Figure 4 shows the evolution of the validation accuracy of MobileNetV2. Validation accuracies for AlexNet and VGG19 are depicted in Figures S2 and S4. Figure 5 shows the confusion matrix of MobileNetV2, the best-performed model. Confusion matrices of AlexNet and VGG19 are depicted in Figures S3 and S5.

### 4.4. Model Testing Using Adversarial Samples

In point-of-care applications of dielectrophoresis, an accurate correlation of pearl chain formation to input voltage can eventually be used to predict the permittivity changes of a microparticle. Unlike images acquired using microscopes, the quality of images obtained in a point-of-care setting may be low. It is likely that the images encounter quality distortions stemming from several artifacts during image acquisition, which can cause the model to fail. The models were tested with adversarial samples, which are micrographs that are of lower quality. Hence, we performed sets of testing with low-quality images to determine the distortion level at which level the performance begins to decrease, so as to identify the models that were invariant to low-quality images. We introduced different types and levels of distortions that degraded image quality.

In order to check the accuracy of the algorithm, we introduced distortions in the original images using ImageJ software. Three types of modifications were introduced to the original images, noise introduction, image blurring, and contrast variation (Figure S6). Noisy images were created by adding zero-mean Gaussian random noise with specified noise variance. We introduced a specified noise of 2%, 5%. 7%, 9%, 10%, 12%, and 15% on images. Image blurring was achieved by introducing Gaussian blur with varying standard deviation (σ) values, which determined the degree of smoothing. σ values of 0.2, 0.5, 0.7, 0.9, 1, 1.2, and 1.5 were used. Contrast variations were obtained by changing the contrast values of original images by 10%, 20%, 50%, 70%, and 90%.

The effect of image quality was significant in predictions. The model was tolerant to contrast variations up to 90%. It also accurately made predictions for images with a 2–10% specific noise level. Nonetheless, a 15% noise gave a reduced prediction accuracy of 0.7. For noise levels >20%, the model was unable to make any accurate predictions. Blurred images with standard deviation values ranging from 0.2 to 1.2 were accurately predicted by our model. However, for values of σ higher than 1.5, the model was unable to make accurate predictions. Our results indicate that the proposed model is susceptible to blur and noise distortions while resilient to contrast variations. In the future, the model will be trained with low-quality images taken under different experimental conditions in order to enhance the accuracy.

## 5. Conclusions

In this work, a low-voltage DEP system was combined with deep learning to estimate DEP forces applied to microparticles. For point-of-care applications of dielectrophoresis, accurate correlation of pearl chain formation to input voltage can be used to predict the intrinsic characteristics of a microparticle. By creating a model that analyzed the micrographs of pearl chains, we showed how deep learning could be used to predict DEP forces exerted on polystyrene (PS) microbeads. Three pretrained CNN architectures were examined. The results from the experiments showed that MobileNetV2 with RMSPROP gave the best performance, with a validation accuracy of 99.29%. Our model was tolerant to contrast variations up to 90%, 80% blurring, and 15% noise level when tested with adversarial samples. The results indicate that the proposed model is robust under unfavorable real-world settings, demonstrating that it can be used as direct dielectrophoretic force estimation in point-of-care settings.

## Figures and Tables

**Table 1:** CNN configuration parameters.

Parameter	Value
$\mathrm{Batch}\;\mathrm{size}\;(m_{b})$	10
Learning rate(α)	0.0001
Epochs	100
Validation frequency	30
Execution environment	CPU

**Table 2:** Definition of optimization algorithms.

Algorithm	Update Rule	Characteristics
RMSProp	$V_{dW}=\;\beta V_{dW}+\left(1-\beta \right)dW^{2}$ $W=W-\alpha \frac{dW}{\sqrt{V_{dW}+\varepsilon }}$	(i) For a given batch size, it utilizes more memory than SGDM but less than ADAM. (ii) This normalizes the effect of decay on the learning rate. (iii) It regulates per-parameter learning rates.

**Table 3:** Performance comparison between the pretrained CNN models.

CNN Model	Optimizer	Validation Accuracy (%)	Training Time Elapsed (s)
AlexNet	RMSPROP	98.57	10880
MobileNetV2	RMSPROP	99.29	27792
VGG19	RMSPROP	97.86	91974

## Supplementary Materials

The following supporting information can be downloaded at: https://www.mdpi.com/article/10.3390/mi13010041/s1, Figure S1: Convolutional neural network architecture; Figure S2: Training progress showing the validation accuracy of AlexNet; Figure S3: Confusion matrix of AlexNet; Figure S4: Training progress showing the validation accuracy of VGG19. Figure S5: Confusion matrix of VGG19; Figure S6: Training progress showing the validation accuracy of MobileNetV2; Figure S7: Confusion matrix of MobileNetV2; Table S1: Performance result of MobileNetV2 on adversarial samples using 7v Dataset; Table S2: Performance result of MobileNetV2 on adversarial samples using 5v Dataset; Table S3: Performance result of MobileNetV2 on adversarial samples using 3v Dataset.
