# Supplementary material for: Deep-Learning Based Estimation of Dielectrophoretic Force
Source: Micromachines (Basel). 2021 Dec 28;13(1):41. doi: 10.3390/mi13010041 (PMC8779967; doi:10.3390/mi13010041)
Supplement: Supplementary file 1 [file micromachines-13-00041-s001.zip › micromachines-1525738-supplementary.pdf]

Supplementary Material

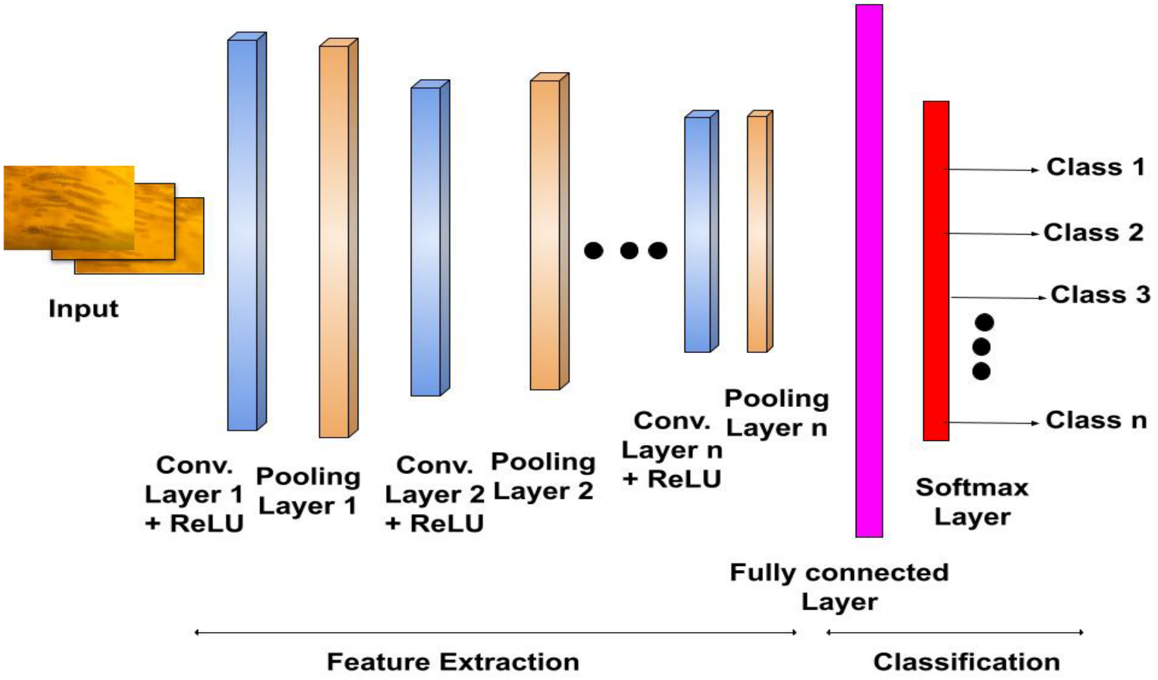

Figure S1: Convolutional neural network architecture.

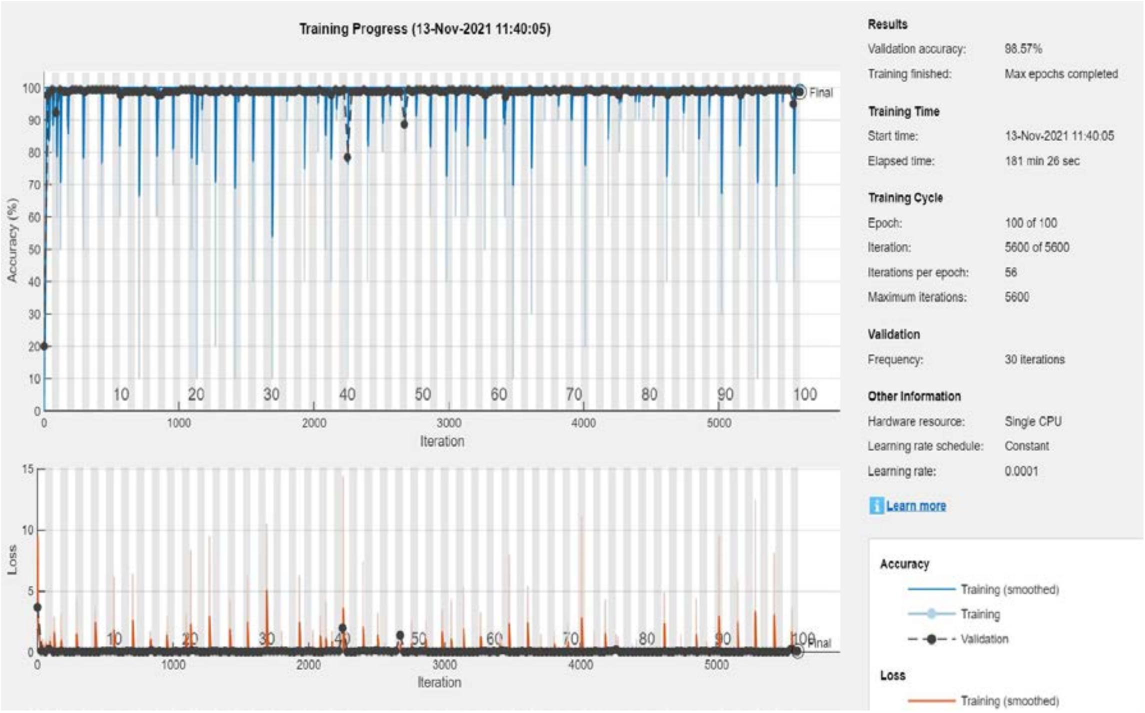

Figure S2: Training progress showing the validation accuracy of AlexNet.

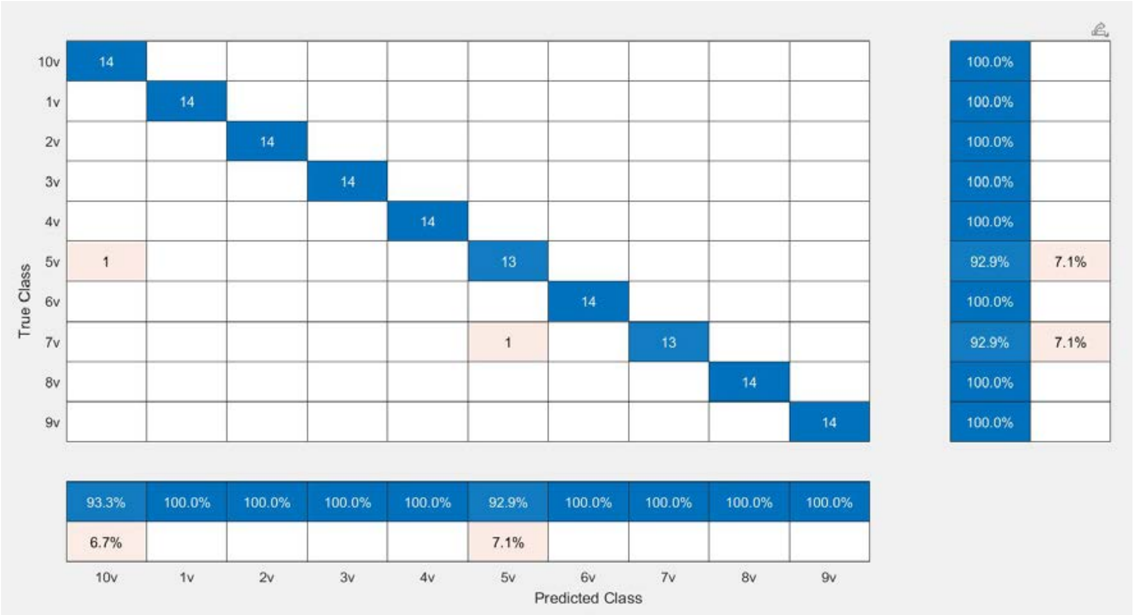

Figure S3: Confusion matrix of AlexNet.

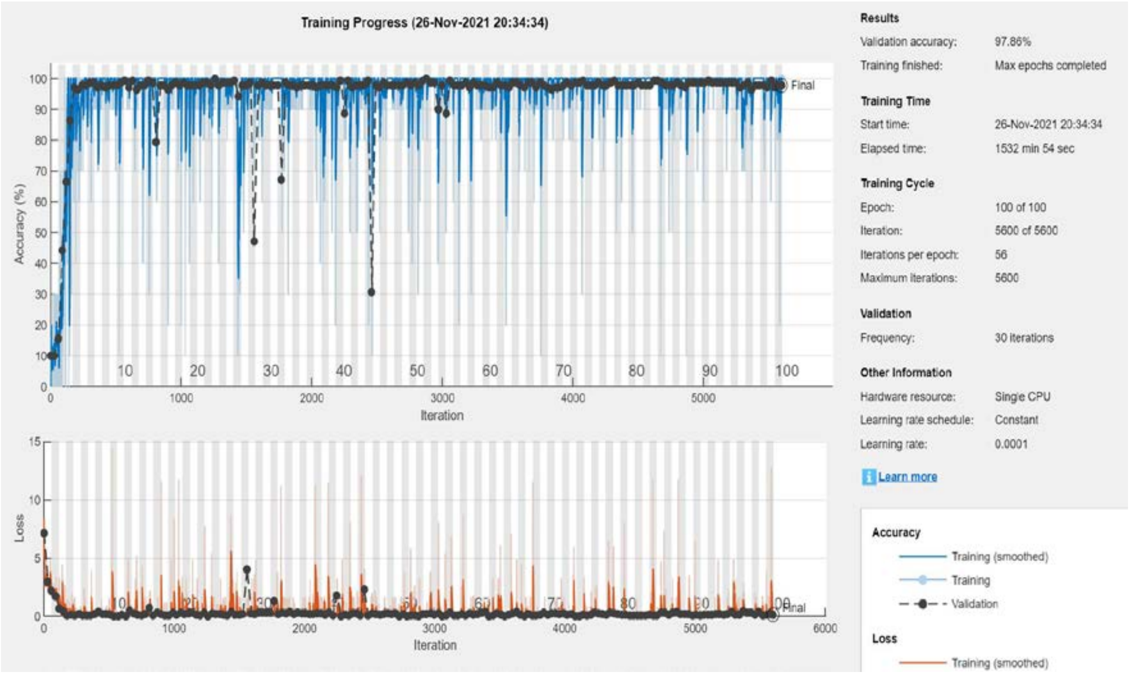

Figure S4: Training progress showing the validation accuracy of VGG19.

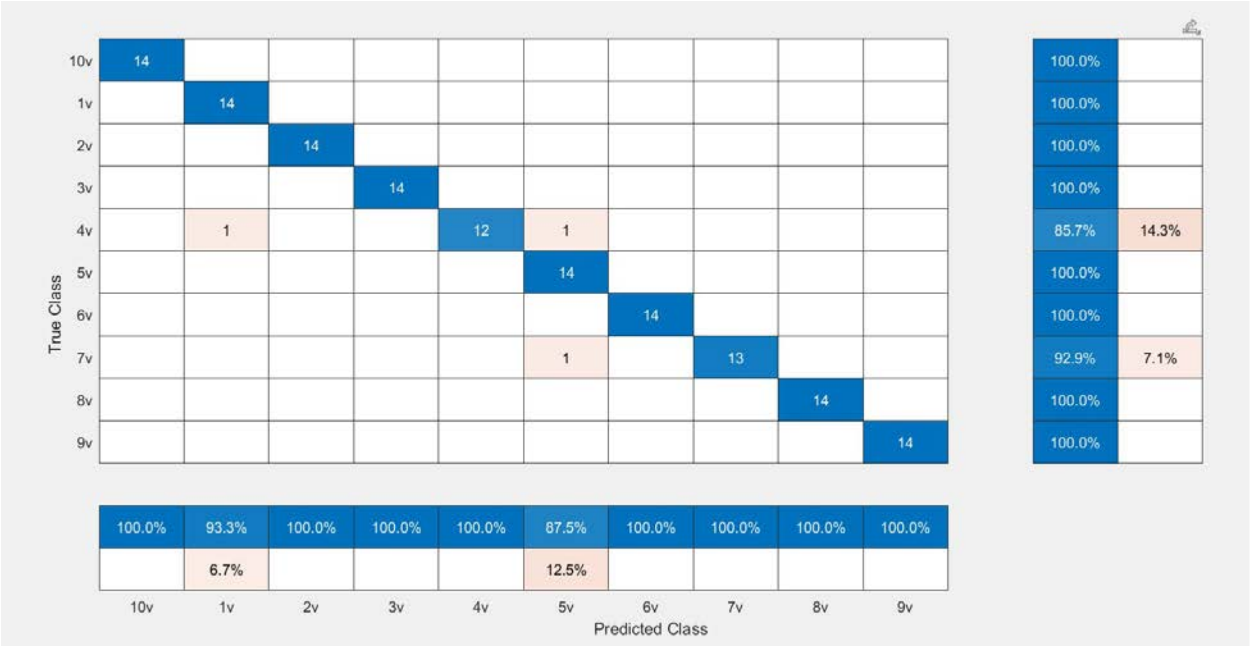

Figure S5: Confusion matrix of VGG19.

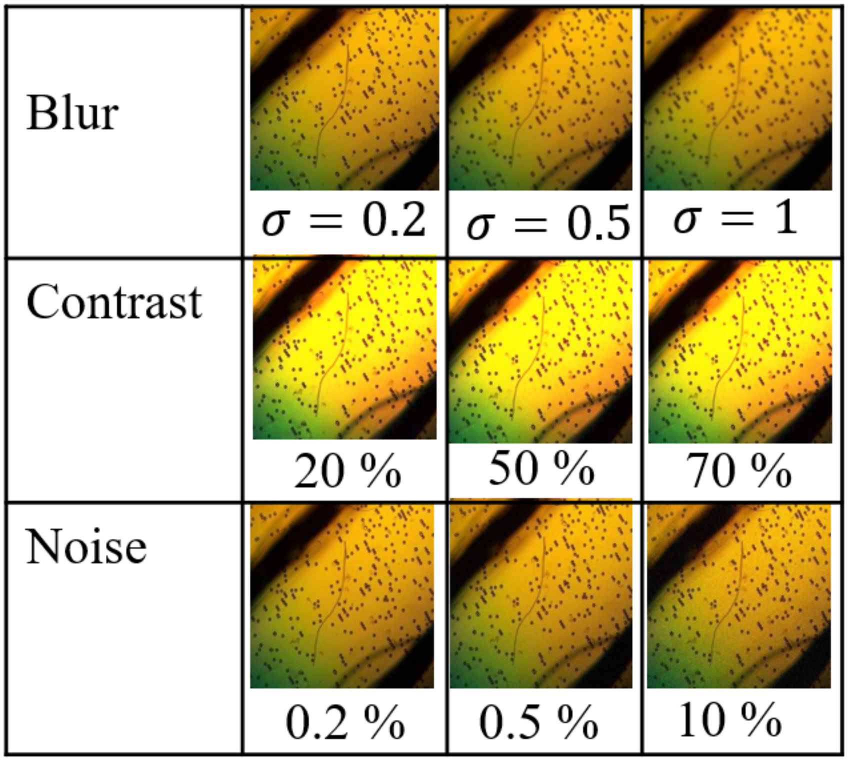

Figure S6: Examples of adversarial sample images with varying Blur, contrast and, noise levels

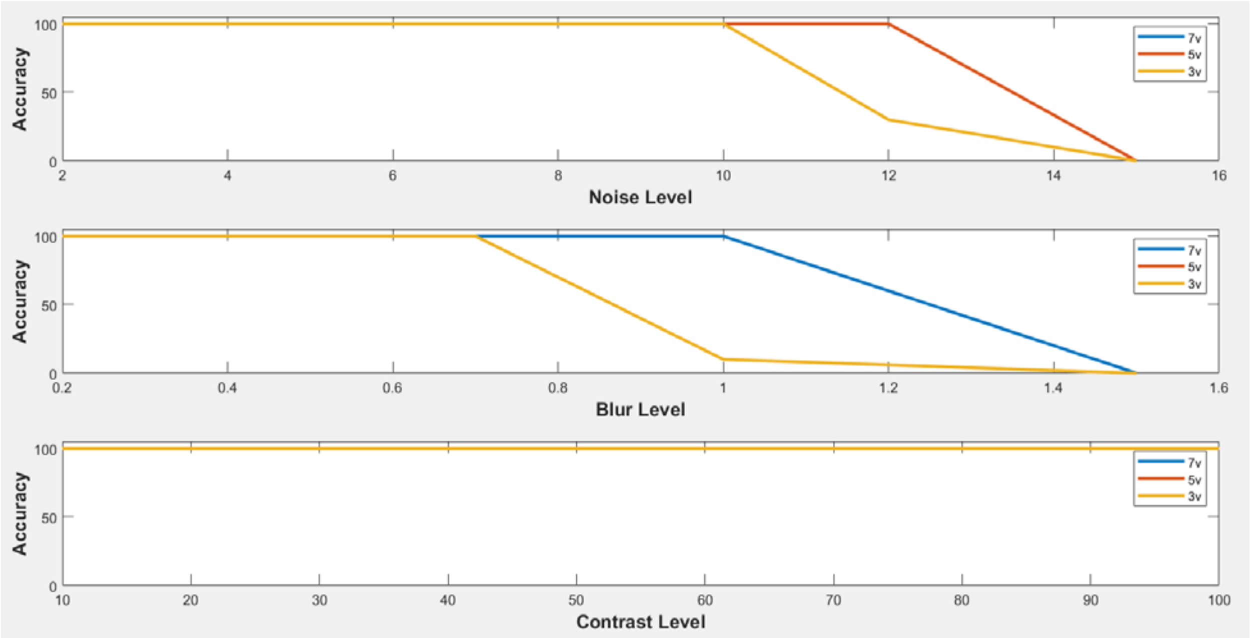

Figure S7: Performance of MobileNetV2 on Adversarial Sample.

Table S1: Performance result of MobileNetV2 on adversarial samples using 7 V Dataset

Images		Number	Accuracy
Original		5/5	100%
Noise	2%	5/5	100%
	5%	5/5	100%
	7%	5/5	100%
	10%	5/5	100%
	12%	5/5	100%
	15%	0/5	0%
Contrast	10%	5/5	100%
	20%	5/5	100%
	50%	5/5	100%
	70%	5/5	100%
	90%	5/5	100%
	100%	5/5	100%
Blur	Value 0.2	5/5	100%
	Value 0.5	5/5	100%
	Value 0.7	5/5	100%
	Value 1	5/5	100%
	Value 1.5	0/5	0%

Table S2: Performance result of MobileNetV2 on adversarial samples using 5 V Dataset

Images		Number	Accuracy
Original		5/5	100%
Noise	2%	5/5	100%
	5%	5/5	100%
	7%	5/5	100%
	10%	5/5	100%
	12%	3/5	30%
	15%	0/5	0%
Contrast	10%	5/5	100%
	20%	5/5	100%
	50%	5/5	100%
	70%	5/5	100%
	90%	5/5	100%
	100	5/5	100%
Blur	Value 0.2	5/5	100%
	Value 0.5	5/5	100%
	Value 0.7	5/5	100%
	Value 1	1/5	10%
	Value 1.5	0/5	0%

Table S3: Performance result of MobileNetV2 on adversarial samples using 3 V Dataset

Images		Number	Accuracy
Original		5/5	100%
Noise	2%	5/5	100%
	5%	5/5	100%
	7%	5/5	100%
	10%	5/5	100%
	12%	5/5	100%
	15%	0/5	0%
Contrast	10%	5/5	100%
	20%	5/5	100%
	50%	5/5	100%
	70%	5/5	100%
	90%	5/5	100%
	100%	5/5	100%
Blur	Value 0.2	1/5	100%
	Value 0.5	0/5	100%
	Value 0.7	0/5	100%
	Value 1	0/5	10%
	Value 1.5	0/5	0%
